# Bioprospecting Underutilized Plant By-Products for Antioxidant Natural Extracts: A Review

**DOI:** 10.3390/molecules31071209

**Published:** 2026-04-06

**Authors:** Jesús Morales-Jiménez, Rosy G. Cruz-Monterrosa, Monzerrat Rosas Espejel, Ildefonso Guerrero-Encinas, Javier N. González-González, Luis Quihui-Cota, Jorge L. Mejía-Méndez, Alejandra Miranda-Carrazco, José E. Aguilar-Toalá

**Affiliations:** 1 Departamento de Ciencias Ambientales, División de Ciencias Biológicas y de la Salud, Universidad Autónoma Metropolitana, Unidad Lerma. Av. de las Garzas 10. Col. El Panteón, Lerma de Villada 52005, Estado de México, Mexico; j.morales@correo.ler.uam.mx; 2 Departamento de Ciencias de la Alimentación, División de Ciencias Biológicas y de la Salud, Universidad Autónoma Metropolitana, Unidad Lerma. Av. de las Garzas 10. Col. El Panteón, Lerma de Villada 52005, Estado de México, Mexico; r.cruz@correo.ler.uam.mx (R.G.C.-M.); m.rosas@correo.ler.uam.mx (M.R.E.); 3 Centro de Investigación en Alimentación y Desarrollo, A.C. (CIAD A.C.), Carretera Gustavo Enrique Astiazarán Rosas No. 46, Col. La Victoria, Hermosillo 83304, Sonora, Mexico; iguerrero221@estudiantes.ciad.mx (I.G.-E.); jgonzalez221@estudiantes.ciad.mx (J.N.G.-G.); lquihui@ciad.mx (L.Q.-C.); 4 Tecnológico de Monterrey, Escuela de Ingeniería y Ciencias, Epigmenio González 500, San Pablo, Santiago de Querétaro 76130, Querétaro, Mexico; mejia.jorge@tec.mx

**Keywords:** by-product valorization, oxidative stress, polyphenols

## Abstract

Underutilized plant by-products are an overlooked source of natural extracts that contain antioxidant bioactive compounds and therapeutic potential. Oxidative stress significantly contributes to the development of various chronic diseases. In this context, natural extracts rich in bioactive compounds derived from underutilized plant by-products emerge as promising options for developing antioxidant-based therapies that target oxidative stress-related molecular pathways involved in the pathogenesis of chronic disease. The valorization of by-products through the recovery of antioxidant-rich extracts is particularly appealing, as non-edible plant parts often contain higher levels of bioactive compounds than their edible counterparts. This review provides a comprehensive overview of antioxidant natural extracts and their major bioactive components, including polyphenols (particularly flavonoids and phenolic acids), terpenoids, alkaloids, and other redox-active compounds.

## 1. Introduction

Many vegetables, fruits, grains, and cereals generate discarded parts (e.g., peels, seeds, stems, leaves, husks, and roots) during agricultural production, food processing and transformation, or when consumed as part of the diet (food wastes), which present an opportunity to valorize underutilized plant by-products. This generation of by-products arises from multiple factors, such as inadequate harvest timing, overproduction, limited use of agricultural products, unfavorable climatic conditions, inefficient harvesting and storage practices, and scarce post-harvest handling. However, many of these plant resources remain underexploited due to insufficient botanical characterization, limited knowledge of their nutritional value, leading to an underestimation of their potential applications, scarce research on their commercial prospects, and low levels of promotion and dissemination [1]. Discarded materials are a valuable source of bioactive compounds, especially natural antioxidants like polyphenols, carotenoids, and bioactive peptides, which can be recovered and used in food, pharmaceutical, and cosmetic industries [2].

In this context, worldwide food losses are estimated to be nearly 50%, occurring at similar rates in both industrialized and developing countries. In developing regions, losses mainly occur after harvest and during processing stages, whereas in industrialized regions, they occur at the retail and consumer levels [3,4]. The global population is expected to increase to 9.8 billion by 2050 and to continue rising to 10.4 billion by the 2080s, intensifying pressure on food systems, natural resources, and environmental sustainability [5].

In this sense, bioprospection of underutilized plant by-products emerges as a sustainable biotechnological approach to address these challenges by turning waste materials into valuable sources of bioactive products. The use of by-products through the recovery of antioxidant-rich extracts is particularly captivating, given that non-edible (discarded) plant parts commonly contain higher levels of bioactive compounds than their edible counterparts. The recovery and potential application of antioxidant-rich extracts from plant by-products contribute to the development of naturally derived ingredients, reduce dependence on synthetic additives, and support circular bioeconomy approaches within agro-food systems [6]. Moreover, the bioprospecting of these by-products aligns with global sustainability goals, particularly Sustainable Development Goal 12 (Responsible consumption and production) of the Food and Agriculture Organization of the United Nations (FAO), by promoting waste reduction, enhancing resource efficiency, and advancing innovation in the development of functional food and nutraceuticals. Relatedly, the concept of a circular economy, often called a “zero-waste” approach, highlights the reutilization of by-products as valuable primary resources, thereby fostering eco-innovation [7]. Consequently, further research on the identification, extraction, characterization, and use of antioxidant compounds from underutilized plant by-products is essential to building more sustainable food systems.

Oxidative stress plays a key role in the development of numerous chronic diseases, including cardiovascular diseases, metabolic syndrome, neurodegenerative diseases, and cancer [8]. In this context, natural antioxidant extracts derived from underutilized plant by-products are promising candidates for modulating redox-sensitive molecular pathways involved in disease pathogenesis [9]. Therefore, advancing research on the identification, extraction, characterization, and biological activity of antioxidant compounds from these plant by-products is essential to support the development of sustainable food systems and novel health-promoting strategies.

This review aims to provide a comprehensive overview of underutilized plant by-products as sources of natural antioxidant extracts, highlighting their major bioactive constituents, extraction methods, mechanisms of antioxidant action, and potential applications in food, pharmaceutical, and nutraceutical industries. To accomplish this, data were compiled from Google Scholar, PubMed, and Web of Science Core Collection (Clarivate Analytics, Philadelphia, PA, USA) databases between December 2025 and March 2026. Original scientific studies (excluding review articles, book chapters) dating from 2020 to 2026 were included. The main search terms used were the “underutilized” OR “underexploited” OR “underused”, AND “plant by-products” OR “agro-industrial residues” OR “food processing waste” OR “agricultural side streams”, AND “bioactive compounds” OR “antioxidant extracts”, consulting scientific studies published in English. The collected literature was screened by examining the titles, abstracts, and keywords, and studies unrelated to the rationale of the review were excluded.

## 2. Underutilized Plant By-Products as Sources of Antioxidant Bioactive Compounds

In recent years, the pharmaceutical and food industries have increasingly focused on the use of natural products rather than synthetic alternatives, driven by growing consumer demand for products derived from natural sources perceived as safe, along with the stricter regulatory frameworks and heightened concerns regarding the toxicological and health implications associated with synthetic compounds [10]. Plant by-products generated during agricultural production, food processing, and consumption encompass a wide range of matrices that are biochemically rich and structurally diverse. Their phytochemical composition is highly dependent on plant species, environmental conditions, cultivar, agronomic practices, maturity stage, and processing conditions, which collectively influence both the qualitative and quantitative profiles of antioxidant compounds [7].

Several studies [11,12,13,14,15] have shown that underutilized plant by-products often contain higher concentrations of antioxidant compounds and exhibit greater antioxidant activity than their edible counterparts [16]. These antioxidant properties are mainly associated with a broad range of phytochemicals, including phenolic acids, flavonoids, tannins, carotenoids, tocopherols, and other bioactive compounds. For example, it has been reported that kiwi peel, compared to kiwi pulp, contains up to twice the phenolic content [13], along with greater levels of tocopherols and organic acids, and higher antioxidant activity [15]. Likewise, the peel of six citrus fruit species demonstrated superior antioxidant capacity and higher concentrations of phytochemicals, particularly total phenolic and flavonoid contents, compared with the pulp [11]. Similarly, seeds and peels from cantaloupe melon display higher total phenolic and antioxidant activity, with peels contributing nearly one-third of the total phenolic content [14]. As reported for other materials of plant origin, the phenolic content in peels (papaya, passion fruit, and pomegranate) is roughly double that of the corresponding seeds and pulp [17].

The preferential accumulation of antioxidant compounds in plant by-products is closely linked to the physiological and ecological roles of secondary metabolites in plant defense systems. External tissues, such as peels, seed coats, and husks, are directly exposed to ultraviolet radiation, pathogens, oxidative stress, and mechanical damage, which promote the biosynthesis and accumulation of protective antioxidant compounds. These molecules play a vital role in alleviating oxidative damage and enhancing plant resilience under adverse environmental conditions [18]. Consequently, plant by-products are particularly attractive matrices for bioprospecting initiatives aimed at recovering high-value antioxidant compounds with potential applications in the food, nutraceutical, pharmaceutical, and cosmetic industries.

## 3. Major Classes of Antioxidant Compounds Identified in Plant By-Products

Plant by-products generated during the processing of fruits and vegetables are increasingly recognized as important sources of antioxidant phytochemicals, in which different major classes of antioxidant compounds have been identified. These compounds, originally associated with the edible portions of plant foods, are often retained or even concentrated in non-edible fractions such as peels, seeds, pomace, and other processing residues. Their chemical diversity and biological activity support the growing interest in valorizing plant by-products for food, nutraceutical, and functional ingredient applications.

Table 1 summarizes the bioactive antioxidant compounds and their reported concentrations in various fruit and vegetable by-products. The compiled data highlight the presence of bioactive antioxidant compounds in various fruit and vegetable by-products, including peels, seeds, pomace, stalks, pods, hearts, rinds, and mixed residues from both temperate and tropical plant species, highlighting the presence of phenolic compounds, (including flavonoids, tannins, anthocyanins), carotenoids, and ascorbic acid in these underutilized matrices. As shown in Table 1, fruit and vegetable by-products exhibit a diverse and often concentrated profile of antioxidant phytochemicals. In particular, fruit peels and seeds (e.g., avocado, Spanish plum, peach, nectarine, mango, acerola, banana, and pomegranate) consistently appear as phenolic- and flavonoid-rich fractions, often surpassing the edible pulp in antioxidant density. In addition, specific compounds such as procyanidins, catechin, and epicatechin have been individually quantified in avocado by-products, highlighting the structural diversity of these extracts. Meanwhile, vegetable residues, including broccoli stalks, cabbage hearts, zucchini and eggplant peels, green bean pods, cucumber peel, and parsnip peel, also retain substantial antioxidant potential, demonstrating that non-fruit matrices can serve as valuable sources of bioactive phytochemicals. Altogether, the updated evidence reinforces the concept that underutilized plant by-products represent concentrated reservoirs of antioxidant compounds. Their compositional richness and variability, depending on species, cultivar, and by-product fraction (peel, seed, pomace, or rind), underscore their relevance for bioprospecting strategies aimed at developing functional ingredients.

Phenolic compounds are secondary plant metabolites that enhance the sensory and nutritional quality of fruits and vegetables, affecting aroma, taste, flavor, and color. Polyphenols are among the most abundant and extensively studied antioxidants found in plant by-products. Within this group, phenolic acids, a major subclass of polyphenols, include hydroxybenzoic and hydroxycinnamic acids (such as gallic, caffeic, ferulic, and p-coumaric acids), which are commonly found in cereal bran, fruit peels, and vegetable residues. These compounds provide antioxidant activity mainly through hydrogen atom donation, metal chelation, and regulation of oxidative enzymes. They are characterized by an aromatic ring substituted with one or more hydroxyl groups, and their structures range from simple phenolic molecules to complex high–molecular-weight polymers. The antioxidant activity of phenolic compounds is strongly structure-dependent, particularly influenced by the number and position of hydroxyl groups and the nature of substitutions on the aromatic rings [37].

Consistent with the data summarized in Table 1, fruit and vegetable by-products such as avocado, Spanish plum, peach, nectarine, mango, acerola, pomegranate, banana, apple, black plum, and grape peels and seeds show particularly high total phenolic contents, often exceeding those reported for edible tissues. In several cases, these fractions also present elevated levels of flavonoids, tannins (both condensed and hydrolysable), and specific compounds such as procyanidins, catechin, and epicatechin. Moreover, certain matrices such as pomegranate peel and tomato pomace stand out for their notable anthocyanin and carotenoid contents (e.g., β-carotene and lycopene). Similarly, vegetable residues, including broccoli stalks, cabbage hearts, zucchini and eggplant peels, cucumber peel, carrot peel and pomace (black and orange varieties), parsnip peel, and green bean pods, contain significant phenolic and flavonoid concentrations, and in some cases relevant carotenoid levels, emphasizing their potential as sustainable sources of antioxidant extracts.

On the other hand, flavonoids, including flavones, flavanols, and anthocyanins, are major contributors to the antioxidant capacity of plant-derived by-products. These compounds are especially abundant in fruit skins and seeds, where they play key physiological roles related to pigmentation, UV protection, and oxidative stress tolerance. As shown in Table 1, high flavonoid levels have been reported in by-products such as avocado peels and seeds, peach and nectarine peels, and various vegetable residues. In several cases, flavonoids are expressed as catechin, quercetin, or rutin equivalents, reflecting methodological differences but consistently indicating a substantial accumulation of these compounds in external tissues and processing fractions. In contrast, anthocyanin-rich by-products, including cashew apple peel, black carrot peel, pomegranate peel, and other pigmented fruit residues, have attracted increasing attention due to their strong radical scavenging activity and their association with potential health benefits related to inflammation and cardiovascular protection. The presence of these compounds in processing residues further emphasizes the functional value of colored plant by-products. Terpenoids, particularly carotenoids, represent another important class of antioxidant compounds found in plant by-products. These lipid-soluble molecules, including β-carotene and lycopene, exert antioxidant activity mainly through singlet oxygen quenching and peroxyl radical scavenging. According to the studies compiled in Table 1, carotenoids are notably present in fruit and vegetable peels such as mango, acerola, carrot, tomato, peach, and cashew apple residues, supporting their potential application in functional foods and nutraceutical formulations.

Although present in lower concentrations than polyphenols, other bioactive components such as alkaloids, saponins, phytosterols, and bioactive peptides may also contribute to the overall antioxidant capacity of certain plant by-products, either directly through redox mechanisms or indirectly by modulating endogenous antioxidant defense systems. In addition, the coexistence of carotenoids with phenolic antioxidants in several matrices suggests potential synergistic effects, supporting their application as multifunctional ingredients in functional foods.

It is important to note that the concentrations reported in Table 1 were determined under different analytical conditions and on different expression bases across studies. Some authors expressed results on a dry weight basis, others on fresh weight, lyophilized material, or per volume of extract (e.g., mg/mL), and in certain cases per gram of extract rather than per gram of raw by-product. Additionally, variations in extraction solvents, solid-to-solvent ratios, drying procedures, and spectrophotometric standards (e.g., gallic acid, catechin, quercetin, or rutin equivalents) may influence the reported values. Therefore, the data presented should be interpreted primarily as indicative of relative antioxidant richness rather than as directly comparable quantitative measurements. Future studies would benefit from methodological harmonization or normalization approaches to enable more robust cross-study comparisons.

## 4. Extraction and Recovery Technologies for Antioxidant Compounds

Extraction is a key separation process used to isolate bioactive compounds from plant by-products through suitable solvents and standard procedures. This method allows for the efficient recovery of soluble components, producing crude extracts that are typically complex mixtures of plant molecules and metabolites such as phenolic compounds (e.g., flavonoids, anthocyanins, and tannins) and carotenoids. These extracts are widely applied in medicinal applications, including as tinctures and liquid extracts [38]. Today, various extraction techniques are available for effectively recovering antioxidant compounds from agro-industrial plant by-products, many of which have been adapted for industrial-scale and commercial use (Table 2).

### 4.1. Conventional Extraction Methods

Traditional extraction methods, such as maceration, Soxhlet extraction, and percolation with organic solvents, are still widely used because they are simple and scalable. However, these techniques often require lengthy extraction times, high solvent consumption, and elevated temperatures, which may compromise the stability of thermolabile antioxidant compounds [39].

In this context, maceration extraction is one of the most widely used conventional methods for obtaining bioactives from plant materials. This method includes soaking the plant matrix in a solvent for extended periods, allowing the diffusion-driven release of target compounds. Polar solvents, such as methanol, ethanol, or their combination with water in various proportions, are commonly used, especially for extracting polyphenols [40]. Although maceration is simple to perform and cost-effective, it is characterized by long extraction times, sometimes lasting several weeks, and high solvent consumption, which can limit its overall efficiency. Compared to other conventional techniques like Soxhlet extraction, which can achieve higher yields through continuous solvent cycling but still require long processing times and large solvent volumes, maceration is generally less efficient and less suitable for intensive or large-scale applications [39].

On the other hand, Soxhlet extraction is a conventional technique regarded as one of the most effective methods for the continuous extraction of bioactives from plant raw materials using a heated solvent. Except for applications involving thermolabile compounds, Soxhlet extraction generally shows better performance compared with other traditional extraction methods. In this process, finely powdered or ground plant material is placed inside a porous thimble, usually made of cellulose or glass fiber, which retains solid particles and prevents their transfer into the extract, thus enabling efficient compound recovery and partial purification. The Soxhlet apparatus consists of a round-bottom flask containing the solvent, an extraction chamber holding the thimble, and a reflux condenser. During operation, the solvent is heated to reflux, its vapors rise through the side arm, condense in the condenser, and percolate through the solid matrix within the thimble. The extracted solutes are then siphoned back into the solvent reservoir. This cycle is repeated multiple times with fresh solvent until the extraction is complete, ensuring exhaustive extraction of the target compounds [39,41].

In this context, Linhares Sabino et al. [53] investigated the recovery of phenolic compounds from spent coffee grounds (an underutilized agro-industrial by-product) using both dynamic maceration and Soxhlet extraction, finding that Soxhlet with ethanol yielded higher phenolic recoveries (23.85%), while maceration with isopropanol (14.66%) and acetone (19.64% dynamic) also produced significant extract yields. Similarly, Florez Montes et al. [54] reported that both solvent extraction (maceration) and Soxhlet extraction have been applied to fruit wastes (e.g., grape peel, soursop peel, mango peel, and grape seed), revealing notable differences in extraction yields and phenolic recoveries between methods. Specifically, the authors found that Soxhlet extraction was more efficient for phenolic compound recovery, yielding 64.65% and 52.28% for grape and mango peels, respectively. In contrast, maceration of the same matrices resulted in lower yields of 61.89% and 38.66%, respectively. These findings are particularly relevant for fruit and vegetable by-products such as avocado peel and seed, pomegranate peel, mango peel, grape pomace, and tomato pomace (Table 1), which contain high concentrations of phenolics and flavonoids. The selection of the extraction method significantly influences recovery efficiency and, consequently, the feasibility of industrial valorization of these matrices.

In contrast, percolation is a traditional extraction method commonly used to obtain bioactive compounds, especially in the preparation of tinctures, fluid extracts, and the recovery of target compounds from diverse plant materials. In this process, finely ground plant material is first moistened with an appropriate solvent and left to sit for several hours in a tightly sealed container. The hydrated material is then packed into a percolator, usually a narrow, cone-shaped vessel with open ends, and covered with an additional volume of solvent to create a thin layer above the solid mass. The system is sealed and kept for a prolonged maceration period, after which the percolator’s outlet is opened to allow the extract to drip slowly under a continuous flow of solvent [39,41].

This continuous percolation of fresh solvent through the plant matrix improves mass transfer, leading to higher extraction yields and shorter processing times compared to static maceration. Additional solvent is added as needed until the percolate reaches about three-quarters of the desired volume. Finally, the spent material is pressed, the remaining solvent is added to the collected extract, and the mixture is clarified by filtration or settling and subsequent decanting [42]. Although less commonly emphasized than maceration or Soxhlet extraction, percolation has been used as a conventional solvent flow method for the recovery of antioxidant phenolic compounds from plant matrices. For example, a comparative study conducted by Shabani et al. [55] has shown that percolation can produce higher flavonoid yields and antioxidant activity than static maceration for *Eryngium planum* extracts.

Overall, although Soxhlet extraction generally provides higher recovery yields, it requires prolonged heating and higher solvent consumption, which may increase energy demand and compromise thermolabile compounds such as anthocyanins and certain carotenoids present in colored peels (e.g., pomegranate, black carrot, or tomato pomace). In contrast, maceration, despite lower efficiency, operates under milder conditions and may better preserve sensitive antioxidants. Therefore, the choice of method should balance extraction yield, compound stability, energy input, and environmental impact. In addition, conventional extraction methods remain important baseline technologies for the recovery of antioxidants from underutilized plant by-products due to their simplicity and scalability. However, their efficiency is matrix-dependent and strongly influenced by solvent polarity, extraction time, and temperature. These limitations justify the growing interest in green and intensified extraction strategies discussed in Section 4.2.

### 4.2. Green and Emerging Extraction Technologies

Some limitations of conventional extraction methods, such as long processing times and the extensive use of hazardous solvents, have driven growing interest in developing sustainable alternatives. In this context, green extraction technologies have gained more attention as effective strategies to address these issues. Emerging sustainable extraction methods like ultrasound-assisted extraction (UAE), microwave-assisted extraction (MAE), pressurized liquid extraction (PLE), supercritical fluid extraction (SFE), electric field–based treatments, and enzyme-assisted extraction (EAE) have become particularly promising options. These advanced techniques provide better extraction efficiency and yield, lower solvent consumption, enhanced selectivity, and higher quality of the recovered bioactive compounds compared to traditional methods [43]. Additionally, the use of green solvents such as ethanol, deep eutectic solvents, and water-based systems enhances the sustainability of these processes and supports bioprospecting strategies aligned with circular bioeconomy principles.

UAE uses acoustic energy with solvents to boost the recovery of target compounds from plant materials. The cavitation phenomena generated by ultrasound, characterized by the rapid formation and collapse of bubbles, promote cell wall disruption, improve solvent penetration, and facilitate the release of intracellular components. Understanding the fundamental extraction mechanisms, kinetics, and the effects of sonication on plant matrices is crucial to optimizing the efficiency of UAE [44,45]. For example, Han et al. [56] optimized UAE coupled with enzymatic treatment for phenolic recovery from peach pomace shows significantly enhanced yields (761 mg gallic acid/L) compared with conventional extraction (698.51 mg gallic acid/L). Similarly, Rodríguez-Pérez et al. [57] reported that UAE significantly enhanced the recovery of phenolic compounds from *Moringa oleifera* Lam leaves, yielding 47 mg gallic acid equivalent/g of dry leaf, whereas the conventional extraction method (maceration) achieved only 27 mg gallic acid equivalent/g of dry leaf, under comparable conditions.

In contrast, MAE employs microwave energy to heat polar solvents in direct contact with the solid sample matrix, allowing efficient recovery of target compounds. During MAE, electromagnetic radiation is transformed into thermal energy mainly through two simultaneous mechanisms: dipole rotation and ionic conduction, which operate synergistically within the solvent–sample system to promote rapid and uniform heating. The effectiveness of this energy conversion depends heavily on the dielectric properties of the solvent, especially the dielectric constant and dissipation factor, which determine the medium’s ability to absorb microwave energy and convert it into heat. Mechanistically, MAE occurs in three sequential stages: desorption of bioactive compounds from the active sites of the sample matrix, internal diffusion of the solvent into the matrix, and external diffusion involving the transfer of solutes from the matrix into the surrounding solvent [46,47]. In this context, MAE was employed (Şen Arslan [36]) using water as the extraction solvent to recover phenolic and antioxidant compounds from red beet, dragon fruit, and prickly pear peels. The resulting extracts exhibited high total phenolic content (ranging from 345.93 to 1651.17 mg gallic acid equivalent/L) and significant antioxidant capacity, demonstrating the potential of MAE for recovering bioactive compounds from vegetable processing residues. Likewise, Jaouhari et al. [58] reported that optimized MAE of raspberry pomace resulted in high yields of total phenolics (68 mg gallic acid equivalent/g of sample) and total flavonoids (63 mg of rutin equivalents/g of sample) with notable bioactivity compared to conventional methods (29.52 mg gallic acid equivalent/g of sample and 18.18 mg of rutin equivalents/g of sample, respectively).

Similarly, PLE is a modern technique for extracting analytes from solid matrices that uses organic solvents at elevated temperatures (around 40–200 °C) and high pressures (500–3000 psi), exceeding the solvents’ normal boiling points. In this method, the solid sample is packed into a stainless-steel extraction cell and subjected to short extraction times (5–15 min), after which the extract is pushed into a collection vial using compressed gas. Meanwhile, SFE has become an appealing alternative for recovering natural products, including polyphenols, because it can operate at relatively low temperatures, consumes less energy, and produces high-quality extracts with improved purity and higher concentrations of target compounds. This technique relies on fluids maintained above their critical temperature and pressure, most commonly carbon dioxide, owing to its low critical point, non-toxicity, non-flammability, cost-effectiveness, and ease of recycling. By adjusting pressure and temperature, the density and solvating power of supercritical CO_2_ can be precisely tuned, enabling selective extraction; however, its application is generally limited to compounds with low to medium polarity, often requiring the addition of organic modifiers, which shifts the process toward subcritical conditions [3,48,49]. In this sense, Domínguez-Rodríguez et al. [59] reported the release of non-extractable polyphenols from cherry pomace via PLE combined with EAE. The authors found that extracts obtained through the combined PLE–EAE approach exhibited substantially higher total phenolic content (75 mg gallic acid equivalent/100 g of sample) compared to extracts derived from conventional methods (8.30 mg gallic acid equivalent/100 g of sample). Similarly, a recent study (Nieto et al. [60]) investigated sustainable PLE conditions to extract antioxidant phenolics from grape seed by-products, optimizing factors such as solvent composition, temperature, and extraction time, and obtaining extracts with high phenolic content (350.80 mg gallic acid equivalent/g of extract) and strong antioxidant activity.

Despite its sustainability and green credentials, SFE remains a technically complex process, a challenge that is further amplified when combined with ultrasound-assisted supercritical fluid extraction (UASFE) due to the multiple interactions among operational variables. Therefore, systematic evaluation and optimization of key parameters are essential to maximize extraction efficiency while minimizing solvent use and energy consumption [17,38,50,51].

Overall, green and emerging extraction technologies are powerful and sustainable tools for bioprospecting underutilized plant by-products as sources of antioxidant natural extracts. Techniques such as UAE, MAE, PLE, and SFE enable more efficient, selective, and environmentally friendly recovery of bioactive compounds while preserving their functional integrity and reducing solvent and energy demands. Although challenges related to process complexity and parameter optimization remain, especially for advanced systems like SFE and UASFE, their integration with green solvents and process intensification strategies strongly supports their use within circular bioeconomy frameworks. Consequently, these technologies are set to play a central role in valorizing plant by-products and promoting the sustainable production of high-value antioxidant ingredients.

In addition, the compositional variability summarized in Table 1 further highlights the need for selecting extraction technologies according to the chemical nature of the target compounds and the reported expression basis. For instance, highly polar compounds such as total phenolics, polyphenols (including flavonoids and tannins), and ascorbic acid, widely reported in avocado, pomegranate, Spanish plum, banana, grape, and vegetable residues, are commonly recovered using hydroalcoholic solvents under conventional or assisted extraction systems. In contrast, lipophilic antioxidants such as carotenoids (e.g., β-carotene and lycopene), identified in mango, carrot, tomato pomace, peach, and apple by-products, require non-polar or moderately non-polar solvents and are particularly suitable for technologies such as supercritical CO_2_ extraction. Moreover, the presence of thermolabile compounds, including anthocyanins in pigmented peels and pomace fractions, reinforces the importance of mild processing conditions and emerging green technologies (e.g., UAE, MAE under controlled parameters, or SFE) to preserve bioactivity. Therefore, the wide range of antioxidant classes and concentration units reported in Table 1 not only reflects compositional richness but also underscores the necessity of optimizing extraction parameters according to matrix type, compound polarity, and intended application.

## 5. Mechanisms of Antioxidant Action and Modulation of Oxidative Stress

Antioxidant compounds derived from plant by-products achieve their biological effects through multiple complementary mechanisms, which are closely related to their chemical makeup and concentration [52]. As demonstrated by various fruit and vegetable by-products, peels, seeds, pomace, and stalks are especially rich in phenolic compounds, (e.g., flavonoids and tannins), carotenoids, and vitamin C, all of which contribute to their strong antioxidant potential (Table 1).

Phenolic compounds such as catechin, epicatechin, procyanidins, caffeoylquinic acids, and hydrolyzable and condensed tannins, widely reported in avocado (*Persea americana*) peels and seeds, Spanish plum (*Spondias purpurea*) by-products, and apple and stone fruit residues, are highly effective at directly scavenging reactive oxygen and nitrogen species (ROS/RNS) and inhibiting lipid peroxidation through hydrogen atom or electron donation. Additionally, their strong metal-chelating capacity limits the availability of pro-oxidant transition metals, thereby reducing radical generation via Fenton-type reactions [61,62].

Carotenoids like β-carotene, especially abundant in cashew apple, acerola, carrot, mango, and strawberry residues, contribute to antioxidant defense by quenching singlet oxygen and stabilizing peroxyl radicals. Meanwhile, L-ascorbic acid, found at high levels in acerola and strawberry by-products, improves redox homeostasis through rapid electron transfer reactions and synergistic interactions with polyphenols [63].

At the cellular level, these bioactive compounds modulate endogenous antioxidant defenses by activating redox-sensitive signaling pathways. Polyphenol-rich extracts from avocado, Brassica vegetables, and fruit pomace have been associated with the activation of the nuclear factor erythroid 2-related factor 2 (Nrf2) pathway, which leads to the upregulation of phase II detoxifying and antioxidant enzymes, including heme oxygenase-1 (HO-1), glutathione S-transferases, and NAD(P)H quinone oxidoreductase 1 [64,65]. At the same time, several phenolic fractions have shown the ability to inhibit nuclear factor kappa B (NF-κB) signaling, reducing oxidative stress–induced inflammation and strengthening the interplay between antioxidant and anti-inflammatory mechanisms [66].

**Figure 1:**
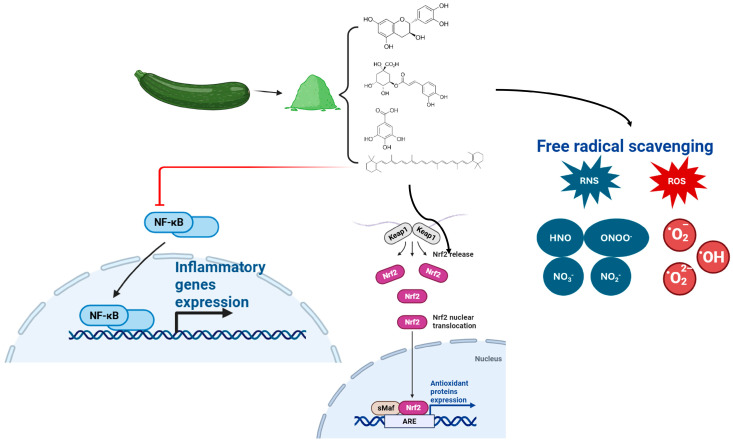
Mechanisms of antioxidant action and modulation of oxidative stress of compounds associated with underutilized plant by-products. Black arrows indicate activation or induction of biological processes, whereas the red T-bar arrow indicates inhibition. The first black arrow represents the generation of bioactive compounds from the plant material.

Overall, the high diversity and concentration of antioxidant compounds present in underutilized plant by-products highlight their importance as functional ingredients capable of modulating oxidative stress at both molecular and cellular levels (Figure 1). These mechanisms support their value in sustainable food, nutraceutical, and pharmaceutical applications, aligning antioxidant effectiveness with circular economy and waste reduction strategies.

In this sense, beyond their high phytochemical content, several plant by-products summarized in Table 1 also exhibit strong antioxidant capacity when evaluated using radical scavenging and reducing power assays. This antioxidant activity was summarized in Table 3 because not all studies report the antioxidant capacity of the extract obtained from plant by-products. The antioxidant capacity reported for extracts obtained from plant by-products varies widely depending on the botanical source, the by-product fraction, and the extraction conditions. Overall, high antioxidant activity is frequently associated with matrices previously reported in Table 1 that contain elevated levels of total phenolic compounds and specific phenolic subclasses, such as flavonoids and phenolic acids. For example, by-products derived from fruit peels, seeds, and pomaces, particularly those from grapes, berries, citrus fruits, and pomegranate, consistently exhibit strong radical scavenging or reducing capacities in assays such as DPPH, ABTS, FRAP, and ORAC. These results are consistent with the phenolic profiles described in Table 1, where these matrices showed high concentrations of compounds such as gallic acid, catechins, quercetin derivatives, chlorogenic acid, and proanthocyanidins, which are well known for their electron-donating and free-radical-quenching properties [67].

However, comparisons across studies should be interpreted with caution because antioxidant activity values were reported using different analytical assays and units (e.g., µM or µg, Trolox or ascorbic acid equivalents, percentage inhibition), which limit direct quantitative comparisons. Despite this variability, a general positive relationship between phenolic concentration and antioxidant capacity is evident. Studies reporting higher total phenolic content values typically correspond to extracts with greater radical scavenging or reducing activity, supporting the widely accepted role of phenolic compounds as the major contributors to the antioxidant potential of plant by-products. Nevertheless, deviations from this trend are also observed in some cases, suggesting that factors such as the presence of other bioactive constituents (e.g., carotenoids, vitamin C, flavonoids), extraction efficiency, and the specific phenolic composition can influence the overall antioxidant response. Collectively, these findings highlight that underutilized plant by-products represent promising sources of natural antioxidants, while also emphasizing the need for standardized analytical approaches to improve cross-study comparability and facilitate their valorization in food, nutraceutical, and functional ingredient applications.

On the other hand, evidence from in vivo models further supports these mechanisms. Polyphenol-rich extracts from plant materials have been shown to improve oxidative stress markers in animal models by increasing antioxidant enzyme activity and reducing lipid peroxidation levels, highlighting their potential therapeutic relevance. For instance, Zhou et al. [68] found that defatted walnut kernel extract and whole walnut kernel extract showed antioxidant properties in vitro and in vivo. The authors found that, in a D-galactose-induced aging mouse model, both extracts significantly increased the activity of key antioxidant enzymes (i.e., superoxide dismutase, catalase, and glutathione peroxidase) and total antioxidant activity, while decreasing lipid peroxidation in the serum and tissue samples, suggesting a protective effect against oxidative stress. Likewise, Figueras et al. [69] found that grape pomace extract attenuated obesity-induced nephropathy in mice fed a high-fat diet by modulating energy metabolism dysregulation and oxidative stress. The authors demonstrated that grape pomace extract protected kidney function by regulating signaling pathways associated with energy metabolism (p-AMPK/p-ACC), inflammation (NLRP3/caspase-1/NF-κB/TNF-α) and oxidative stress, including reduced oxidant species production and downregulation of Nox4. Similarly, in a recent study by Han et al. [70] reported that extracts from grape seed and grape peels alleviated obesity in high-fat diet mice and improved gut microbiota composition as well as short-chain fatty acids production.

## 6. Potential Applications in Food, Pharmaceutical, Cosmeceutical, and Nutraceutical Industries

The diverse range and high concentrations of bioactive compounds found in underutilized plant by-products highlight their strong potential for use in the food, pharmaceutical, and nutraceutical industries. Fruit and vegetable residues like peels, seeds, pomace, stalks, and pods, which are often discarded during industrial processing, are concentrated sources of phenolic compounds (such as flavonoids and tannins), carotenoids, and vitamins. As shown by by-products from avocado, apple, carrot, mango, Spanish plum, acerola, Brassica vegetables, and stone fruits, these matrices provide multifunctional ingredients capable of providing antioxidant, anti-inflammatory, and cytoprotective effects [71].

In the food industry, extracts rich in polyphenols (particularly flavonoids) from by-products such as apple peel and pomace, avocado residues, carrot peels, and cabbage and broccoli fractions show significant potential as natural antioxidants for enhancing oxidative stability and extending the shelf life of lipid-rich foods. Carotenoid-rich residues, including carrot, mangoes, cashew apples, and acerola by-products, further offer technological advantages as natural colorants with added antioxidant properties, supporting clean-label formulations and substituting synthetic additives [72].

From a nutraceutical and pharmaceutical perspective, the high levels of catechins, epicatechins, procyanidins, hydrolyzable and condensed tannins, and vitamin C found in avocado seeds, Spanish plum, acerola, strawberries, and stone fruit by-products support their use in functional foods, dietary supplements, and complementary therapeutic formulations. These compounds have been associated with the modulation of oxidative stress and inflammation through redox-sensitive pathways such as Nrf2 and NF-κB, emphasizing their importance in preventing and managing chronic diseases associated with oxidative imbalance [25,73].

Beyond their antioxidant properties, bioactive compounds derived from plant by-products have attracted increasing attention for their potential role in the prevention or management of chronic diseases associated with oxidative stress and inflammation. In particular, obesity and related metabolic disorders represent major global health challenges where nutraceutical interventions may provide complementary benefits. Several experimental studies have demonstrated that polyphenol-rich extracts obtained from fruit by-products can modulate metabolic and inflammatory pathways. For example, grape pomace polyphenols have been shown to alleviate high-fat-diet–induced obesity in mice by improving gut microbiota composition and increasing short-chain fatty acid production, thereby contributing to metabolic regulation [70]. Similarly, supplementation with grape pomace extract has been reported to mitigate obesity-related metabolic alterations, including insulin resistance, lipid accumulation, and inflammatory signaling, while protecting organ function by modulating pathways such as AMPK, NF-κB, and oxidative stress-related mechanisms [69].

These findings suggest that plant by-product–derived extracts, particularly those rich in phenolic compounds, could serve as promising nutraceutical ingredients for improving metabolic health and reducing inflammation-associated diseases. However, further clinical studies are required to confirm their efficacy, bioavailability, and long-term safety in humans.

Despite these promising applications, several challenges continue to hinder the full industrial and commercial exploitation of bioactive extracts from plant by-products. Variability in raw material composition, influenced by cultivar, maturity, plant growing conditions, and processing methods, complicates standardization and reproducibility. Additionally, the lack of optimized and harmonized extraction protocols, along with difficulties in maximizing yields during scale-up of green and emerging extraction technologies, remains a significant obstacle for industrial implementation. Limited in vivo and clinical evidence, combined with regulatory constraints related to safety assessment, efficacy validation, and approval for use in food and nutraceuticals, further limit market adoption [39].

In addition to food and nutraceutical applications, plant by-products generated by the agro-food industry have gained increasing attention as sustainable ingredients in cosmetic and cosmeceutical formulations. These residues are often rich in polyphenols, carotenoids, vitamins, and essential oils that exhibit antioxidant, anti-inflammatory, antimicrobial, and photoprotective properties beneficial for skin health. Extracts from fruit peels and seeds, such as banana, pomegranate, citrus, and grape, have been incorporated into cosmetic formulations, including cleansing gels, creams, shampoos, and sunscreens. For example, polyphenol-rich extracts from banana and pomegranate peels have demonstrated antioxidant and antimicrobial activities and have been used to improve the functional properties of cleansing gels and other skincare products [34]. Similarly, citrus by-products are an important source of bioactive compounds, such as hesperidin and essential oils, which exhibit anti-inflammatory, antioxidant, and antimicrobial effects that can contribute to anti-aging formulations, skin barrier protection, and the treatment of hyperpigmentation and UV-induced damage [74]. Furthermore, other fruit processing residues, such as mango peel, have been successfully incorporated into cosmetic products (e.g., solid shampoo formulations), where their phenolic compounds enhance antioxidant stability and may serve as natural alternatives to synthetic additives [75].

The valorization of plant by-products as cosmetic ingredients aligns with circular economy principles and the growing demand for natural and sustainable cosmetic formulations. These materials represent promising sources of functional bioactive compounds capable of improving skin protection, delaying photoaging, and enhancing the stability of cosmetic products.

Despite growing interest in valorizing plant by-products as sources of bioactive compounds, several practical considerations must be addressed to enable their large-scale industrial implementation. From an economic perspective, the feasibility of producing antioxidant extracts depends on factors such as raw material availability, extraction efficiency, processing costs, and downstream purification requirements [76,77]. Many extraction technologies currently remain at intermediate technology readiness levels (TRL 3–5), meaning that although promising results have been demonstrated at laboratory and pilot scales, further optimization and scale-up are required for full industrial deployment [78]. In addition, regulatory frameworks represent an important challenge. In the European Union, the approval of new food ingredients must comply with the regulations established by the European Food Safety Authority (EFSA), while in the United States, ingredients intended for food or nutraceutical use must often achieve Generally Recognized as Safe (GRAS) status under the U.S. Food and Drug Administration (FDA) [79,80]. These processes require extensive toxicological evaluation, safety validation, and standardized characterization of bioactive extracts. Furthermore, logistical aspects of supply chains, including seasonal availability of raw materials, compositional variability, and transportation and storage conditions, may influence the consistency and scalability of production. Another critical factor is the stability of bioactive compounds during storage and processing, as phenolic compounds, carotenoids, and vitamins may degrade under exposure to light, oxygen, temperature, and moisture [81,82]. Therefore, strategies such as encapsulation, optimized packaging systems, and controlled storage conditions are often necessary to preserve antioxidant activity and ensure product shelf life [83]. Addressing these technological, regulatory, and economic challenges will be essential to facilitate the successful commercialization of plant by-product–derived bioactive ingredients.

Future research should therefore prioritize integrative and multidisciplinary approaches combining advanced analytical tools, bioactivity-guided fractionation, omics-based technologies, and life-cycle assessments to enhance extraction efficiency, stabilize bioactive compounds, and promote sustainability. Building stronger collaborations among academia, industry, and policymakers will be crucial to harmonize regulatory frameworks and facilitate the translation of laboratory-scale findings into safe, scalable, and cost-effective solutions [52]. Overall, these efforts will support the sustainable valorization of underutilized plant by-products and speed up their incorporation into next-generation food, pharmaceutical, and nutraceutical products.

## 7. Conclusions

Underutilized plant by-products are a promising and sustainable source of natural antioxidant extracts with significant potential for food, pharmaceutical, cosmeceutical, and nutraceutical applications. Advances in bioprospecting strategies, together with the development of green extraction technologies, have facilitated the efficient recovery of bioactive compounds from these materials and improved our understanding of their mechanisms of action in oxidative stress modulation. These approaches contribute to transforming agro-industrial residues into high-value functional ingredients, supporting circular economy principles and sustainable resource management. However, despite the growing body of laboratory-scale evidence demonstrating the antioxidant potential of these extracts, several challenges remain for their successful translation into commercial products. Issues related to economic feasibility, technological scalability, regulatory approval, supply chain logistics, and the stability of bioactive compounds during processing and storage must be carefully addressed. Future research should therefore focus not only on improving extraction efficiency and bioactivity characterization, but also on advancing pilot-scale validation, standardizing extracts, conducting safety assessments, and ensuring regulatory compliance. Overall, valorizing plant by-products as sources of natural antioxidants is a promising strategy to simultaneously promote human health, reduce food system waste, and support the development of resilient, resource-efficient, and sustainable agro-food systems.

## Figures and Tables

**Table 1:** Bioactive antioxidant compounds and their reported concentrations in a wide range of fruit and vegetable by-products.

Plant Source and By-Product Fraction	Bioactive Compounds and Concentration Reported	References
Cashew apple (*Anacardium occidentale* L.) peel	Anthocyanins (14.74 mg/100 g dry basis)	[19]
	Yellow flavonoids (44.91 mg/100 g dry basis)	
	β-carotene (0.179 mg/100 g dry basis)	
Avocado “Hass” (*Persea americana* Mill.) peel	Total phenolic content (63.5 mg gallic acid equivalent/g of lyophilized)	[20]
	Procyanidin B_2_ (48.38 µg/mg of extract)	
	Epicatechin (40.21 µg/mg of extract)	
Avocado “Hass” (*Persea americana* Mill.) seed	Total phenolic content (57.3 mg gallic acid equivalent/g of lyophilized)	[20]
	Trans-5-O-caffeoyl-D-quinic acid (1.63 µg/mg of extract)	
	Procyanidin B_1_ (1.52 µg/mg of extract)	
	Catechin (3.64 µg/mg of extract)	
	Epicatechin (10.27 µg/mg of extract)	
Avocado “Fuerte” (*Persea americana* Mill.) peel	Total phenolic content (120.3 mg gallic acid equivalent/g of lyophilized)	[20]
	Procyanidin B_2_ (28.34 µg/mg of extract)	
	Epicatechin (30.40 µg/mg of extract)	
Avocado “Fuerte” (*Persea americana* Mill.) seed	Total phenolic content (59.2 mg gallic acid equivalent/g of lyophilized)	[20]
	Trans-5-O-caffeoyl-D-quinic acid (5.74 µg/mg of extract)	
	Procyanidin B_1_ (2.27 µg/mg of extract)	
	Catechin (8.13 µg/mg of extract)	
	Epicatechin (11.06 µg/mg of extract)	
Zucchini (*Cucurbita pepo*) peel	Total phenolic content (69.87 mg gallic acid equivalent/g of sample)	[21]
	Total flavonoids (59.06 mg catechin equivalent/g of sample)	
Eggplant (*Solanum melongena*) peel	Total phenolic content (336.3 mg gallic acid equivalent/g of sample)	[21]
	Total flavonoids (32.08 mg catechin equivalent/g of sample)	
Broccoli (*Brassica oleracea* var. *italica*) stalk	Total phenolic content (62.42 mg gallic acid equivalent/g of sample)	[21]
	Total flavonoids (37.68 mg catechin equivalent/g of sample)	
Cabbage (*Brassica oleracea* var. *capitate*) heart	Total phenolic content (63.88 mg gallic acid equivalent/g of sample)	[21]
	Total flavonoids (22.44 mg catechin equivalent/g of sample)	
Green beans (*Phaseolus vulgaris*) pod	Total phenolic content (33.92 mg gallic acid equivalent/g of sample)	[21]
	Total flavonoids (31.14 mg catechin equivalent/g of sample)	
Spanish plum (*Spondias purpurea* L.) peel	Total phenolics (extractable and non-extractable) (5772.35 mg gallic acid equivalent/100 g of dry matter)	[22]
	Hydrolyzable tannins	
Spanish plum (*Spondias purpurea* L.) seed	Total phenolics (extractable and non-extractable) (3917.75 mg gallic acid equivalent/100 g of dry matter)	[22]
Peaches (*Prunus persica*) peels (different varieties)	Total phenolic content (*ca*. 300–450 mg gallic acid equivalent/100 g of sample)	[23]
Nectarines (*Prunus persica* var. *nucipersica*) peels (different varieties)	Total phenolic content (*ca*. 238–350 mg gallic acid equivalent/100 g of sample)	[23]
Acerola (*Malpighia emarginata*) peel	β-carotene (5.12 mg/g of sample dried)	[24]
	L-ascorbic acid (3.06 mg/g of sample dried)	
	Condensed and hydrolysable tannins (3.86 and 28.21 mg/g of sample dried)	
Acerola (*Malpighia emarginata*) seed	β-carotene (3.24 mg/g of sample dried)	[24]
	L-ascorbic acid (26.83 mg/g of sample dried)	
	Condensed and hydrolysable tannins (2.9 and 19.76 mg/g of sample dried)	
Strawberry (*Fragaria* spp.) residue	β-carotene (0.86 mg/g of sample dried)	[24]
	L-ascorbic acid (26.83 mg/g of sample dried)	
	Condensed and hydrolysable tannins (2.12 and 23.55 mg/g of sample dried)	
Papaya (*Carica papaya* L.) peel and seed	Total phenolic content (0.06 and 0.12 mg gallic acid equivalent/mL of extract)	[25]
	Total flavonoid content (0.05 and 0.10 mg quercetin equivalent/mL of extract)	
	Condensed tannins (0.15 and 0.48 mg catechin equivalent/mL of extract)	
Mango (*Mangifera indica* L. var. osteen) peel and seed	Total phenolic content (0.27 and 1.36 mg gallic acid equivalent/mL of extract)	[25]
	Total flavonoid content (0.09 and 0.21 mg quercetin equivalent/mL of extract)	
	Condensed tannins (0.97 and 1.04 mg catechin equivalent/mL of extract)	
Loquat (*Eriobotrya japonica* Thunb.) peel and seed	Total phenolic content (0.23 and 0.30 mg gallic acid equivalent/mL of extract)	[25]
	Total flavonoid content (0.05 and 0.13 mg quercetin equivalent/mL of extract)	
	Condensed tannins (0.34 and 0.82 mg catechin equivalent/mL of extract)	
Apple (*Malus domestica* Borkh) peel and pomace	Total phenolic content (1228 and 47 mg gallic acid equivalent/g of dry weight basis)	[26]
	Total flavonoid content (2434 and 79 mg catechin equivalent/g of dry weight basis)	
	Total carotenoid content (48.2 and 23.9 µg β-carotene equivalent/g of dry weight basis)	
Black carrot (*Daucus carota* L.) peel	Total phenolic content (1934 mg gallic acid equivalent/g of dry weight basis)	[26]
	Total flavonoid content (466 mg catechin equivalent/g of dry weight basis)	
	Total carotenoid content (194 µg β-carotene equivalent/g of dry weight basis)	
Orange carrot (*Daucus carota* L.) peel and pomace	Total phenolic content (495 and 8 mg gallic acid equivalent/g of dry weight basis)	[26]
	Total flavonoid content (20 and 6 mg catechin equivalent/g of dry weight basis)	
	Total carotenoid content (753.2 and 436.3 µg β-carotene equivalent/g of dry weight basis)	
Cucumber (*Cucumis sativus* L.) peel	Total phenolic content (168 mg gallic acid equivalent/g of dry weight basis)	[26]
	Total flavonoid content (25 mg catechin equivalent/g of dry weight basis)	
	Total carotenoid content (7.1 µg β-carotene equivalent/g of dry weight basis)	
Kumbat (*Citrus japonica* Thumb) peel plus pomace	Total phenolic content (561 mg gallic acid equivalent/g of dry weight basis)	[26]
	Total flavonoid content (13 mg catechin equivalent/g of dry weight basis)	
	Total carotenoid content (348.3 µg β-carotene equivalent/g of dry weight basis)	
Mango (*Mangifera indica* L.) peel and pomace	Total phenolic content (1915 and 366 mg gallic acid equivalent/g of dry weight basis)	[26]
	Total flavonoid content (166 and 65 mg catechin equivalent/g of dry weight basis)	
	Total carotenoid content (91.7 and 65 µg β-carotene equivalent/g of dry weight basis)	
Parsnip (*Pastinaca sativa* L.) peel	Total phenolic content (117 mg gallic acid equivalent/g of dry weight basis)	[26]
	Total flavonoid content (5 mg catechin equivalent/g of dry weight basis)	
	Total carotenoid content (5.8 µg β-carotene equivalent/g of dry weight basis)	
Peach (*Prunus persica* L. Batsch) peel and pomace	Total phenolic content (400 and 246 mg gallic acid equivalent/g of dry weight basis)	[26]
	Total flavonoid content (394 and 363 mg catechin equivalent/g of dry weight basis)	
	Total carotenoid content (92.1 and 68.6 µg β-carotene equivalent/g of dry weight basis)	
Black plum (*Prunus domestica* L.) peel and pomace	Total phenolic content (1576 and 938 mg gallic acid equivalent/g of dry weight basis)	[26]
	Total flavonoid content (2396 and 1518 mg catechin equivalent/g of dry weight basis)	
	Total carotenoid content (50.9 and 22.6 µg β-carotene equivalent/g of dry weight basis)	
Grape (*Vitis vinifera* L.) pomace seeds	Total phenolic content (*ca*. 50–100 mg gallic acid equivalent/g of dry weight)	[27]
	Total flavonoid content (*ca*. 40–80 mg catechin equivalent/g of dry weight basis)	
Pomegranate (*Punica granatum* L.) peel	Total phenolic content (60–200 mg gallic acid equivalent/g of dry weight basis)	[28]
	Total flavonoid content (23–85 mg rutin equivalent/g of dry weight basis)	
	Total anthocyanin content (325–985 µg cyanidin 3-O-glucoside equivalent/g of dry weight basis)	
	Ascorbic acid content (5–14 mg/g of dry weight basis)	
Banana (*Musa acuminata* cv. sagor) peel	Total phenolic content (22.95–25.99 mg gallic acid equivalent/g of dry weight basis)	[29]
	Total flavonoid content (13.68–14.30 mg quercetin equivalent/g of dry weight basis)	
Banana (*Musa acuminata* cv. Grande Nain) peel	Total phenolic content (15.97–50.3 mg gallic acid equivalent/g of dry weight basis)	[30]
	Total flavonoid content (18.23–55.4 mg quercetin equivalent/g of dry weight basis)	
	Tannins (19.34–36.05 mg tannic acid equivalent/g of dry weight basis)	
Pomegranate (*Punica granatum* L.) peel	Total phenolic content (172.36 mg gallic acid equivalent/g of dry weight basis)	[31]
Watermelon (*Citrullus lanatus*) peel and rind	Total phenolic content peel (1.76–3.97 mg gallic acid equivalent/g of dry weight basis)	[32]
	Total flavonoid content peel (18.21–73.79 mg quercetin equivalent/100 g of dry weight basis)	
	Total phenolic content rind (1.63–3.57 mg gallic acid equivalent/g of dry weight basis)	
	Total flavonoid content rind (16.62–41.86 mg quercetin equivalent/100 g of dry weight basis)	
Tomato (*Solanum lycopersicum* L.) pomace	Total phenolic content (122.03–134.71 mg gallic acid equivalent/kg of dry weight basis)	[33]
	Total carotenoids content (2.24–4.24 mg β-carotene equivalent/g of dry weight basis)	
	β-carotene (2.95–3.14 µg/g of dry weight basis)	
	Lycopene (6.10–7.41 µg/g of dry weight basis)	
Tomato (*Solanum lycopersicum* L.) pomace	Total phenolic content (10.99 mg gallic acid equivalent/100 mL extract)	[34]
Banana (*Musa* sp.) peel	Total phenolic content (16.54 mg gallic acid equivalent/100 mL extract)	[34]
Pomegranate (*Punica* sp.) peel	Total phenolic content (16.52 mg gallic acid equivalent/100 mL extract)	[34]
Grape (*Vitis* sp.) pomace	Total phenolic content (13.44 mg gallic acid equivalent/100 mL extract)	[34]
Spent coffee (*Coffea Arabica* L.) grounds	Total phenolic content (41.6–53.7 mg gallic acid equivalent/100 g of dry weight basis)	[35]
Red beet (*Beta vulgaris* L.)	Total phenolic content (936.84 mg gallic acid equivalent/L extract)	[36]
Prickly pear (*Opuntia ficus-indica*)	Total phenolic content (1651.17 mg gallic acid equivalent/L extract)	[36]
Dragon fruit (*Hylocereus undatus*)	Total phenolic content (345.93 mg gallic acid equivalent/L extract)	[36]

**Table 2:** Advantages and disadvantages of extraction and recovery technologies for antioxidant compounds [39,40,41,42,43,44,45,46,47,48,49,50,51,52].

Extraction Method	Advantages	Disadvantages
**Conventional methods**		
Maceration	Easy to implement Cost-effective	Extensive extraction times Extreme solvent consumption
Soxhlet extraction	High extraction efficiency Reduce solvent consumption	Time-consuming Unsuitable for thermolabile compounds
Percolation	High-yield extraction Low cost and simplicity Suitability for thermally unstable compounds	Time-consuming High solvent usage
**Green and emerging methods**		
Ultrasound-assisted extraction	High efficiency and speed Lower use of solvent	Scalability issues Free radical formation Potential degradation
Microwave-assisted extraction	High yields Reduced solvent usage Speed	Thermal degradation High cost and scale-up issues
Pressurized liquid extraction	High efficiency Speed Reduce solvent consumption	High cost Degradation of thermolabile compounds Sample pre-treatment
Supercritical fluid extraction	Environmental and safety benefits Useful for thermolabile compounds No residual solvents Reduced cost and time	High investment cost High operational cost Technical complexity Limited scale
Electric field-based extraction	High efficiency and yield No thermal degradation Lower solvent use Speed	High cost Limited applications Complex optimization
Enzyme-assisted extraction	High efficiency and yield Gentle processing conditions Improved product quality Reduced energy consumption	High cost Time-consuming Complex optimization

**Table 3:** Antioxidant capacity reported for extracts from plant by-products.

Plant By-Product	Antioxidant Capacity and Antioxidant Assay	Reference
Avocado “Hass” (*Persea americana* Mill.) peel	310 µM Trolox equivalents/g of sample lyophilized (DPPH) 791.5 µM Trolox equivalents/g of sample lyophilized (ABTS) 1175.1 µM Fe^2+^/g of sample lyophilized (FRAP)	[20]
Avocado “Hass” (*Persea americana* Mill.) seed	410.7 µM Trolox equivalents/g of sample lyophilized (DPPH) 645.8 µM Trolox equivalents/g of sample lyophilized (ABTS) 656.9 µM Fe^2+^/g of sample lyophilized (FRAP)	[20]
Avocado “Fuerte” (*Persea americana* Mill.) peel	420.5 µM Trolox equivalents/g of sample lyophilized (DPPH) 1004.5 µM Trolox equivalents/g of sample lyophilized (ABTS) 1881.4 µM Fe^2+^/g of sample lyophilized (FRAP)	[20]
Avocado “Fuerte” (*Persea americana* Mill.) seed	464.9 µM Trolox equivalents/g of sample lyophilized (DPPH) 580.8 µM Trolox equivalents/g of sample lyophilized (ABTS) 931.7 µM Fe^2+^/g of sample lyophilized (FRAP)	[20]
Zucchini (*Cucurbita pepo*) peel	159.13 µM Trolox equivalents/100 g of sample (DPPH)	[21]
Eggplant (*Solanum melongena*) peel	250.94 µM Trolox equivalents/100 g of sample (DPPH)	[21]
Broccoli (*Brassica oleracea* var. *italica*) stalk	223.10 µM Trolox equivalents/100 g of sample (DPPH)	[21]
Cabbage (*Brassica oleracea* var. *capitate*) heart	198.87 µM Trolox equivalents/100 g of sample (DPPH)	[21]
Green beans (*Phaseolus vulgaris*) pod	168.53 µM Trolox equivalents/100 g of sample (DPPH)	[21]
Peaches (*Prunus persica*) peels (different varieties)	*ca*. 6000–9500 µM Trolox equivalents/100 g of freeze-dried sample (ABTS) *ca*. 5500–7500 µM Trolox equivalents/100 g of freeze-dried sample (ORAC) *ca*. 3000–4000 µM Trolox equivalents/100 g of freeze-dried sample (DPPH)	[23]
Nectarines (*Prunus persica* var. *nucipersica*) peels (different varieties)	*ca*. 4500–6500 µM Trolox equivalents/100 g of freeze-dried sample (ABTS) *ca*. 5000–8000 µM Trolox equivalents/100 g of freeze-dried sample (ORAC) *ca*. 2100–3000 µM Trolox equivalents/100 g of freeze-dried sample (DPPH)	[23]
Acerola (*Malpighia emarginata*) peel	143.45 µM Trolox equivalents/g of dry weight basis (DPPH) 125.01 µM Trolox equivalents/g of dry weight basis (ABTS) 787.91 µM Trolox equivalents/g of dry weight basis (FRAP) 70.98 µM Trolox equivalents/g of dry weight basis (ORAC)	[24]
Acerola (*Malpighia emarginata*) seed	20.39 µM Trolox equivalents/g of dry weight basis (DPPH) 52.63 µM Trolox equivalents/g of dry weight basis (ABTS) 162.96 µM Trolox equivalents/g of dry weight basis (FRAP) 34.08 µM Trolox equivalents/g of dry weight basis (ORAC)	[24]
Strawberry (*Fragaria* spp.) residue	29.41 µM Trolox equivalents/g of dry weight basis (DPPH) 105.41 µM Trolox equivalents/g of dry weight basis (ABTS) 112.05 µM Trolox equivalents/g of dry weight basis (FRAP) 19.39 µM Trolox equivalents/g of dry weight basis (ORAC)	[24]
Apple (*Malus domestica* Borkh) peel and pomace	4.95 and 0.73 µg Trolox equivalents/mg of dry weight basis (DPPH)	[26]
Black carrot (*Daucus carota* L.) peel	2.64 µg Trolox equivalents/mg of dry weight basis (DPPH)	[26]
Orange carrot (*Daucus carota* L.) peel and pomace	3.31 and 0.14 µg Trolox equivalents/mg of dry weight basis (DPPH)	[26]
Cucumber (*Cucumis sativus* L.) peel	0.33 µg Trolox equivalents/mg of dry weight basis (DPPH)	[26]
Kumbat (*Citrus japonica* Thumb) peel plus pomace	3.26 µg Trolox equivalents/mg of dry weight basis (DPPH)	[26]
Mango (*Mangifera indica* L.) peel and pomace	5.64 and 2.38 µg Trolox equivalents/mg of dry weight basis (DPPH)	[26]
Parsnip (*Pastinaca sativa* L.) peel	0.36 µg Trolox equivalents/mg of dry weight basis (DPPH)	[26]
Peach (*Prunus persica* L. Batsch) peel and pomace	2.25 and 1.77 µg Trolox equivalents/mg of dry weight basis (DPPH)	[26]
Black plum (*Prunus domestica* L.) peel and pomace	3.17 and 4.30 µg Trolox equivalents/mg of dry weight basis (DPPH)	[26]
Pomegranate (*Punica granatum* L.) peel	784–2307 µM ascorbic acid equivalents/g of dry weight basis (FRAP) 435–1843 µM ascorbic acid equivalents/g of dry weight basis (DPPH)	[28]
Banana (*Musa acuminata* cv. sagor) peel	79.03–82.50% inhibition (DPPH) 85.52–88.83% inhibition (ABTS)	[29]
Pomegranate (*Punica granatum* L.) peel	276.65 mg Trolox equivalents/g of dry weight basis (DPPH) 357.20 mg Trolox equivalents/g of dry weight basis (ABTS) 527.15 mg Trolox equivalents/g of dry weight basis (FRAP)	[31]
Spent coffee (*Coffea Arabica* L.) grounds	50.6–78.1 mg Trolox equivalents/100 g of dry weight basis (DPPH) 0.5–1.8 mg Trolox equivalents/100 g of dry weight basis (ABTS)	[35]

## Data Availability

Not applicable.
